# Neurocognitive aging data release with behavioral, structural and multi-echo functional MRI measures

**DOI:** 10.1038/s41597-022-01231-7

**Published:** 2022-03-29

**Authors:** R. Nathan Spreng, Roni Setton, Udi Alter, Benjamin N. Cassidy, Bri Darboh, Elizabeth DuPre, Karin Kantarovich, Amber W. Lockrow, Laetitia Mwilambwe-Tshilobo, Wen-Ming Luh, Prantik Kundu, Gary R. Turner

**Affiliations:** 1 Montreal Neurological Institute, Department of Neurology and Neurosurgery, McGill University, Montreal, QC Canada; 2 McConnell Brain Imaging Centre, McGill University, Montreal, QC Canada; 3 Departments of Psychiatry and Psychology, McGill University, Montreal, QC Canada; 4 Douglas Mental Health University Institute, Verdun, QC Canada; 5 Department of Psychology, York University, Toronto, ON Canada; 6 Department of Psychology, Ryerson University, Toronto, ON Canada; 7 Department of Psychiatry, University of Toronto, Toronto, ON Canada; 8 National Institute on Aging, National Institutes of Health, Baltimore, MD USA; 9 Icahn School of Medicine at Mount Sinai, New York, NY USA

**Keywords:** Cognitive neuroscience, Cognitive ageing, Magnetic resonance imaging

## Abstract

Central to understanding human behavior is a comprehensive mapping of brain-behavior relations within the context of lifespan development. Reproducible discoveries depend upon well-powered samples of reliable data. We provide to the scientific community two, 10-minute, multi-echo functional MRI (ME-fMRI) runs, and structural MRI (T1-MPRAGE), from 181 healthy younger (ages 18–34 y) and 120 older adults (ages 60–89 y). T2-FLAIR MRIs and behavioral assessments are available in a majority subset of over 250 participants. Behavioral assessments include fluid and crystallized cognition, self-reported measures of personality, and socioemotional functioning. Initial quality control and validation of these data is provided. This dataset will be of value to scientists interested in BOLD signal specifically isolated from ME-fMRI, individual differences in brain-behavioral associations, and cross-sectional aging effects in healthy adults. Demographic and behavioral data are available within the Open Science Framework project “Goal-Directed Cognition in Older and Younger Adults” (http://osf.io/yhzxe/), which will be augmented over time; neuroimaging data are available on OpenNeuro (https://openneuro.org/datasets/ds003592).

## Abstract (summary)

**Table:** 

Measurement(s)	Magnetization-Prepared Rapid Gradient Echo MRI • Functional Magnetic Resonance Imaging • T2 and Fluid Attenuated Inversion Recovery MRI • Cognition
Technology Type(s)	3 Tesla Magnetic Resonance Imaging • Cognitive Assessment
Sample Characteristic - Organism	Homo sapiens
Sample Characteristic - Location	United States of America • Canada

## Background & Summary

Comprehensive characterization, or deep phenotyping, is critically necessary to identify reliable patterns of coherence between brain structure, function and behavior, towards generating precision maps of brain-behavior associations. Here we report data collected from a two-site, cognitive neuroscience investigation involving samples of healthy younger and older adults. The design was cross-sectional, incorporating deep behavioral phenotyping, structural MRI and multi-echo fMRI (ME-fMRI) data acquisition. The central motivation of the study was to investigate differences across age cohorts in cognition and brain health, and how they intersect to shape late life development. The sample size for each age-cohort recruited into the study was calculated to provide sufficient statistical power for group-wise individual difference analyses of behavior-brain associations^1^. A correlation of individual differences between measures that do not share method variance is, on average, between 0.20 and 0.30^2–4^, and n ≥ 120 provides 80% power to detect non-zero correlations r ≥ 0.20 with 95% confidence intervals.

In devising our behavioral protocol, we drew from research showing that cognitive aging proceeds along two overarching trajectories: fluid cognition steadily declines with age while crystallized cognition increases or remains stable^5^. To obtain reliable behavioral indices we included multiple measures for each cognitive domain including episodic memory, semantic memory, executive functioning and processing speed, as well as self-report measures of personality and socioemotional functioning.

In developing the functional brain imaging protocol, we adopted a lifespan network neuroscience approach^6^. This involves investigating neurocognitive aging through the lens of spatially distributed, large-scale brain networks. With advancing age, the network architecture of the brain shifts, as within-network connectivity declines, between-network connectivity increases, and network dedifferentiation emerges as a prominent feature of the aging connectome (see^7^, for in-depth preliminary examination of the dataset). We reasoned that the dual trajectories of cognitive aging would be reflected in these broad shifts in the functional architecture of the aging brain^8,9^.

Identifying reliable patterns of functional networks and associations with cognition imposes significant methodological challenges. Among the most pervasive of these involves the separation of neural from non-neural, or noise, components in resting-state BOLD signals. This is particularly problematic in cross-sectional studies of aging, where it can be difficult to attribute observed group differences to neural sources versus noise sources of non-interest. We adopted ME-fMRI data acquisition with multi-echo independent components analysis (ME-ICA) preprocessing^10^. This approach relies on the TE-dependence model of BOLD signal for denoising to reliably differentiate BOLD from non-BOLD signal in fMRI data^10^. Importantly, ME-ICA processing removes distant-dependent motion confounds in RSFC data^11^, possibly eliminating the need for multiple confound regression, including the global signal^12^, while allowing for valid between-group comparisons of the full range of positive and negative RSFC values. The approach has proven to be highly reliable for precision mapping^13^. Subsets of the data described here have provided novel insight into inter-regional BOLD signal variability properties in young adults^14^, and into large-scale network configuration differences between younger and older adults related to semantic autobiographical memory^15^ and moral cognition^16^. Despite the demonstrable advantages of ME-fMRI data and processing, there is currently a paucity of ME-fMRI data available in open access repositories (for task and naturalistic viewing exceptions, see^17,18^).

This release is the product of a multi-year, multi-site data collection initiative with the overarching objective to uncover parallels in the shifting architectures of brain and cognitive functioning from younger to older adulthood. In addition to resting-state ME-fMRI, we also collected a structural image (T1-MPRAGE) and a T2-FLAIR acquisition to quantify white matter hyperintensities. Our initial investigation into the older adults included in this dataset suggests that white matter hyperintensities, which accumulate with advancing age, are related to reduced network segregation^19^. This multi-modal protocol has resulted in an extensive neurocognitive aging dataset, including one of the first large-scale releases of ME-fMRI data in younger and older adults. We expect that the samples of healthy younger and older adults reported here, as well as the deep phenotyping and innovative neuroimaging approaches, will be of broad interest to the scientific community.

## Methods

### Participants

Participants were 181 younger (*M*_*age*_ = 22.59 y, *SD* = 3.27; 57% female) and 120 older (*M*_*age*_ = 68.63 y, *SD* = 6.44; 54% female) healthy adults from Ithaca, New York (N = 238) and Toronto, Canada (N = 63; Table 1), rendering a total sample size of 301 that passed quality assessment. Participants were screened to rule out individuals with a history of neurological or other medical illness known to impact cognition, acute or chronic psychiatric illness, those undergoing current or recent treatment with psychotropic medication, and those having recently experienced significant changes to health status at the time of the eligibility interview. Younger and older participants were screened for depressive symptoms using the Beck Depression Inventory^20^ and the Geriatric Depression Scale^21^, respectively. Two older adults were not included due to a rating of “moderate depression.” Participants were additionally administered the Mini-Mental State Examination (MMSE^22^). Participants with MMSE scores below 27/30 were excluded if fluid cognition scores^23^ also fell below an age-adjusted national percentile of 25% (two younger and two older adults). All participants were right-handed with normal or corrected-to-normal vision. Procedures were administered in compliance with the Institutional Review Board at Cornell University and the Research Ethics Board at York University, including written informed consent obtained from each study participant. The cost of data collection per participant was estimated to be approximately $3000 USD.

**Table 1:** Sample Demographics.

	Descriptive Statistics		Inferential Statistics				
	Younger Adults	Older Adults	*T*	dof	*p*	95% CI	Cohen’s *d*
N							
Cornell	154 (86 female, 68 male)	84 (47 female, 37 male)					
York	27 (17 female, 10 male)	36 (19 female, 17 male)					
Race	60.38% White, 19.50% Asian, 8.18% Black, 5.03% other, 4.41% mixed, 2.50% not provided	92.38% White, 2.54% Asian, 2.54% Black, 2.54% other					
Ethnicity	81.76 non-Hispanic or Latino, 10.69% Hispanic or Latino, 7.55% not provided	89.83% non-Hispanic or Latino, 8.48% not provided, 1.69% Hispanic or Latino					
Age (years)							
*Range*	18–34	60–89					
*M*	22.6	68.6					
*SD*	3.3	6.4					
Education (years)*			−7.20	285	<0.001	[−2.5, −1.48]	0.86
*Range*	12–24	12–24					
*M*	15.2	17.2					
*SD*	1.9	2.9					
Episodic Memory*			17.51	281	<0.001	[1.1, 1.38]	2.11
*Range*	−1.75–1.59	−1.99–0.70					
*M*	0.52	−0.71					
*SD*	0.53	0.66					
Semantic Memory*			−9.18	281	<0.001	[−1.00, −0.65]	1.10
*Range*	−2.78–1.39	−1.29–1.91					
*M*	−0.35	0.48					
*SD*	0.77	0.71					
Executive Function*			12.67	281	<0.001	[0.77, 1.02]	1.73
*Range*	-1.16–1.80	−2.03–0.76					
*M*	0.36	−0.48					
*SD*	0.56	0.53					
Processing Speed*			15.03	281	<0.001	[1.17, 1.53]	1.81
*Range*	−2.26–3.05	−2.40-0.050					
*M*	0.57	−0.78					
*SD*	0.86	0.56					

*Note:* Episodic Memory, Semantic Memory, and Executive Function are index scores. Processing Speed is a z-score on Symbol Digit Modalities Task, Oral. * significant group differences. Education was not recorded for 14 participants. Age group differences in episodic memory, semantic memory, executive function, and processing speed were tested in 283 participants. Positive T values reflect higher scores in younger adults, negative values reflect higher scores in older adults. Statistical results were nearly identical when including sex, education, site, and estimated whole brain volume as covariates in an ANCOVA.

### Cognitive, Behavioral and Personality Assessment

In the lab, 283 of 301 individuals (163/181 younger adults, 120/120 older adults) underwent extensive cognitive, behavioral and personality assessment over three to four days prior to brain scanning, and passed quality assessment.

In lab assessments included the NIH Toolboxes of Cognition and Emotion^23^ and auxiliary measures. The NIH Cognition Toolbox included the Rey Auditory Verbal Learning and Picture Sequence Memory, Flanker Inhibitory Control and Attention, Dimensional Change Card Sort, List Sort Working Memory, Picture Vocabulary, and Oral Reading Recognition tests. Composite scores of fluid and crystallized intelligence were also tabulated within the toolbox. The NIH Emotion Toolbox included surveys of Positive Affect, General Life Satisfaction, Meaning and Purpose, Emotional Support, Instrumental Support, Friendship, Perceived Rejection, Perceived Hostility, Perceived Stress, Self-Efficacy, Anger-Affect, Fear-Somatic Arousal, and Fear-Affect. Additionally, participants completed Verbal Paired Associates from the Wechsler Memory Scale-IV^24^, the Associative Recall Paradigm^25^, Shipley-2 Vocabulary^26^, Trail Making Test B-A^27^, the Reading Span Task^28^, and the Symbol Digit Modalities Test^29^.

Online, 253 of 301 individuals (142/181 younger adults, 111/120 older adults) completed self-report evaluations between lab visits in the days prior to brain scanning, and passed quality assessment. Measures included the Behavioral Inhibition System/Behavioral Activation System Scale^30^, Interpersonal Reactivity Index^31^, and Big Five Aspects Scale^32^.

### Magnetic resonance imaging

Neuroimaging data were acquired from two sites with a 3T GE Discovery MR750 and 32-channel head coil at the Cornell Magnetic Resonance Imaging Facility or on a 3T Siemens TimTrio MRI scanner with a 32-channel head coil at the York University Neuroimaging Center in Toronto.

### T1-MPRAGE

T1 anatomical scans on the GE were acquired using a T1-weighted volumetric magnetization prepared rapid gradient echo sequence (TR = 2530 ms; TE = 3.4 ms; 7° flip angle; 1 mm isotropic voxels, 176 slices, 5m25s) with 2x acceleration with sensitivity encoding. On the Siemens, anatomical scans were acquired using a T1-weighted volumetric magnetization prepared rapid gradient echo sequence (TR = 1900ms; TE = 2.52 ms; 9° flip angle; 1 mm isotropic voxels, 192 slices, 4m26s) with 2x acceleration and generalized auto calibrating partially parallel acquisition (GRAPPA) encoding at an iPAT acceleration factor of 2.

### T2-FLAIR

A subset of 110 older adults and 148 younger adults have T2-FLAIR images. T2-weighted FLAIR sequences were acquired on a GE (TR = 12000 ms; TE = 95 ms; TI = 2712 ms; 160° flip angle; 42 slices of 1x1x3 mm; 2m36s) and Siemens (TR = 12000 ms; TE = 95 ms; TI = 2759.4 ms; 160° flip angle; 44 slices of 0.8 × 0.8 × 3 mm; 3m38s). 12 participants had FLAIR images with 46 slices acquired due to technician error, detailed in the README and participants.tsv files on OpenNeuro^33^.

### Resting-state ME-fMRI

All participants completed two 10m06s resting-state multi-echo BOLD functional scans. Participants were instructed to keep their eyes open, blinking and breathing normally in the dimly lit scanner bay. Resting-state runs were acquired using a multi-echo (ME) EPI sequence on GE (TR = 3000 ms; TE_1_ = 13.7 ms, TE_2_ = 30 ms, TE_3_ = 47 ms; 83° flip angle; matrix size = 72 × 72; field of view (FOV) = 210 mm; 46 axial slices; 3 mm isotropic voxels; 204 volumes, 2.5x acceleration with sensitivity encoding) and Siemens (TR = 3000 ms; TE_1_ = 14 ms, TE_2_ = 29.96 ms, TE_3_ = 45.92 ms; 83° flip angle; matrix size = 64 × 64; FOV = 216 mm; 43 axial slices; 3.4 × 3.4 × 3mm voxels; 200 volumes, 3x acceleration and GRAPPA encoding) scanners. One participant (sub-149) had 206 volumes collected instead of 204: This discrepancy is detailed in the README and participants.tsv file on OpenNeuro^33^. For 233 participants scanned on the GE, pulse and respiration were monitored continuously during scanning using an integrated pulse oximeter and respiratory belt. Note that due to a software upgrade, physiological sampling varies between participants at 50 Hz or 40 Hz, as indicated in the Data Records.

### Data collection quality assurance and control

A number of measures were taken to ensure reliable high quality behavioral and neuroimaging data collection.

In lab behavioral data were collected by a trained psychometrist. All behavioral data were then quality controlled before and after compilation. Paper and pencil measures were digitized by two researchers to ensure accuracy. Online data collection included multiple attention checks. Participants with incorrect responses to 4 or more checks were excluded. Participants who answered 3 or more different questionnaires uniformly (i.e., without variation in the Likert ratings) were excluded completely, along with participants missing more than 15% of total behavioral data. Participants missing more than 15% of any given measure were not included in any analysis with that measure.

Prior to undergoing MRI scanning, all participants were informed about the importance of staying still during the MR scan. All scans at the Cornell Magnetic Resonance Imaging Facility and York University Neuroimaging Center were performed by a trained MR technician working with a standardized protocol. This ensured consistent data acquisition procedures, including visual checks for coverage, ongoing quality assessment, and confirmation of participant wakefulness between runs. Five participants were initially excluded after visual inspection of anatomical scans revealed anomalies. One younger participant had a hyperintensity in the posterior lateral ventricle. One younger and four older adults had extended basal ganglia lesions of unknown etiology resembling perivascular spaces^34,35^. As the cognitive consequences of perivascular spaces in cognitively healthy adults remain unknown (e.g.^36,37^), these participants were excluded from our sample. Image quality assessment was then performed on each functional run following preprocessing to exclude participants with unsuccessful coregistration, residual noise (framewise displacement > 0.50 mm coupled with denoised data with DVARS > 1^38^), a temporal signal-to-noise ratio <50, or an insufficient amount of data retained after denoising (<10 BOLD components; see BOLD dimensionality below). Only participants with two functional runs that met these criteria were included in our final sample. 9 younger and 24 older adults were excluded on this basis.

Quality metrics on the final sample are discussed below.

## Data Records

All demographic information, in addition to cognitive, behavioral and personality variables, is available within the Open Science Framework project “Goal-Directed Cognition in Older and Younger Adults” contributed by R.N.S. (10.17605/OSF.IO/YHZXE; http://osf.io/yhzxe/^39^). All neuroimaging data are available on OpenNeuro following Brain Imaging Data Structure (BIDS) specification^40^ along with a detailed descriptor of acquisition parameters (10.18112/openneuro.ds003592.v1.0.8^33^). All data is shared under the Creative Commons license CC0.

### Demographics, Cognitive, Behavioral and Personality Assessment

Location: ddbehav.csv

File format: plain text, comma-separated values

Basic demographic information including gender, age, race, and ethnicity is available.

All demographic information is available as a comma-separated value (CSV) file.

### T1 Anatomical scans

Location: sub- <ID> /ses-1/anat/sub- <ID>_ses-1-T1w.nii.gz

File format: NIfTI, gzip-compressed

MRI data are available in NIfTI file format. All structural scans have been defaced as part of the de-identifying process.

### T2-FLAIR scans

Location: sub- <ID> /ses-1/anat/sub- <ID>_ses-1_FLAIR.nii.gz

File format: NIfTI, gzip-compressed

### Resting-state ME-fMRI

Location: sub- <ID> /ses- <SESSION> /func/sub- <ID>_ses- <SESSION>_task-rest_echo[1-3]_bold.nii.gz

File format: NIfTI, gzip-compressed

### Physiological recordings

Location: sub- <ID> /ses- <SESSION> /func/sub- <ID>_ses- <SESSION>_task-rest_physio.tsv.gz

File format: plain text, tab-separated values

Physiological recordings for respiration and heart rate are provided as a tab-separated value (TSV) file. Accompanying meta-data in JSON format indicates the sampling frequency.

## Technical Validation

### Cognitive, Behavioral and Personality Assessment

Composite scores of episodic memory, semantic memory, and executive function were created from cognitive measures. Missing cells were first imputed with age group means (36 younger adults and 42 older adults had at least one cell missing). Latency scores on the Trail Making Task were reversed so that higher values on all measures reflected better performance. Scores were then z-scored and averaged for each composite. Episodic memory included scores on Verbal Paired Associates, Associative Recall, NIH Cognition Rey Auditory Verbal Learning, and NIH Cognition Picture Sequence Memory; Semantic memory included scores on Shipley Vocabulary, NIH Cognition Picture Vocabulary, and NIH Cognition Oral Reading Recognition; Executive function included scores on the Trail Making Task, NIH Cognition Flanker Inhibitory Control and Attention, NIH Cognition Dimensional Change Card Sort, and NIH Cognition List Sort Working Memory. Processing speed was additionally measured with the Symbol Digits Modalities Task.

Descriptive statistics for all cognitive measures are shown in Table 1. Overall, younger adults had higher episodic memory (*t*(281) = 17.51, *p* < 0.001, [1.10, 1.38], Cohen’s *d* = 2.11), executive function (*t*(281) = 12.67, *p* < 0.001, [0.71, 0.97], Cohen’s *d* = 1.52), and processing speed (*t*(281) = 15.03, *p* < 0.001, [1.17, 1.53], Cohen’s *d* = 1.81) scores than older adults. Older adults had higher semantic memory scores (*t*(281) = 9.18, *p* < 0.001, [−1.00, −0.65], Cohen’s *d* = 1.10) than younger adults. These results remained when controlling for site, gender, and education.

Table 2 contains descriptive statistics for all individual measures included in the index scores. Table 3 contains descriptive statistics for all self-report measures.

**Table 2:** Descriptive Statistics for Cognitive Measures by Age Group (N = 283).

Measure	Younger Adults			Older Adults			*F*	*p* (uncorrected)	ηp2
	Mean	SD	Range	Mean	SD	Range			
*Episodic Memory*									
Verbal Paired Associates: Immediate Recall	43.24	8.11	14.00–55.00	30.52	8.88	11.00–53.00	125.82	<0.001	0.31
Verbal Paired Associates: Delayed Recall	12.99	1.53	7.00–14.00	9.26	2.68	3.00–14.00	192.32	<0.001	0.41
Verbal Paired Associates: Delayed Free Recall	21.14	3.74	9.00–28.00	15.38	4.55	6.00–26.00	109.73	<0.001	0.28
Associative Recall	178.41	42.13	34.00–238.00	110.31	50.89	9.00–219.00	117.13	<0.001	0.30
NIH Cognition Rey Auditory Verbal Learning: Immediate Recall	31.97	5.09	17.00–45.00	23.97	5.11	10.00–34.00	130.65	<0.001	0.33
NIH Cognition Picture Sequence Memory	118.79	13.83	83.66–135.55	98.43	11.22	76.42–135.55	128.19	<0.001	0.32
*Semantic Memory*									
Shipley Vocabulary	32.96	3.77	19.00–39.00	36.11	2.62	27.00–40.00	52.06	<0.001	0.16
NIH Cognition Picture Vocabulary	120.70	10.00	89.44–145.27	134.30	10.10	108.54–153.07	103.64	<0.001	0.28
NIH Cognition Oral Reading Recognition	127.37	10.19	104.89–150.71	132.57	11.24	97.48–150.71	1.69	0.195	0.01
*Executive Function*									
Trail Making Task: B-A	2818.80	1863.82	−422.00–11865.00	3750.69	2640.61	−109.00–15972.00	13.01	<0.001	0.04
NIH Cognition Flanker Inhibitory Control and Attention	106.63	13.95	85.09–142.11	94.12	6.23	82.86–121.18	77.06	<0.001	0.22
NIH Cognition Dimensional Change Card Sort	114.48	10.41	95.40–143.94	99.73	8.22	86.21–123.75	126.08	<0.001	0.32
NIH Cognition List Sort Working Memory	117.85	11.44	93.90–144.50	106.67	9.10	88.68–130.38	56.06	<0.001	0.17
*Processing Speed*									
Symbol Digits Modality	76.02	12.14	36.00–111.00	56.95	7.92	34.00–75.00	155.08	<0.005	0.36

*Note*. NIH Cognition scores are unadjusted. One-way ANCOVAs were conducted on each measure to test for age group differences with site, gender, and education as covariates.

**Table 3:** Descriptive Statistics for Self-Report Measures by Age Group (N = 253).

Measure	Younger Adults			Older Adults			*F*	*p* (uncorrected)	ηp2
	Mean	SD	Range	Mean	SD	Range			
Big Five Aspects Scale: Openness (Aspect)	3.79	0.54	2.30–4.95	3.86	0.54	1.70–4.85	0.00	0.99	0.00
Big Five Aspects Scale: Conscientiousness (Aspect)	3.48	0.56	1.85–4.50	3.65	0.56	2.15–5.00	3.86	0.051	0.02
Big Five Aspects Scale: Extraversion (Aspect)	3.63	0.59	2.20–4.75	3.56	0.65	1.70–4.85	0.42	0.515	0.00
Big Five Aspects Scale: Agreeableness (Aspect)	3.84	0.56	1.50–4.95	4.10	0.43	2.20–4.95	12.59	<0.001	0.05
Big Five Aspects Scale: Neuroticism (Aspect)	2.69	0.68	1.10–4.60	2.23	0.60	1.00–3.95	26.19	<0.001	0.10
Big Five Aspects Scale: Withdrawal (Facet)	2.72	0.74	1.00–4.70	2.16	0.67	1.00–4.20	30.64	<0.001	0.11
Big Five Aspects Scale: Volatility (Facet)	2.67	0.82	1.10–4.50	2.30	0.69	1.00–4.10	12.38	<0.001	0.05
Big Five Aspects Scale: Compassion (Facet)	3.97	0.71	1.50–5.00	4.11	0.55	2.10–5.00	2.13	0.145	0.01
Big Five Aspects Scale: Politeness (Facet)	3.71	0.63	1.50–4.90	4.10	0.48	2.30–5.00	21.88	<0.001	0.08
Big Five Aspects Scale: Industriousness (Facet)	3.47	0.66	2.00–4.90	3.78	0.69	1.90–5.00	9.62	<0.005	0.04
Big Five Aspects Scale: Orderliness (Facet)	3.50	0.66	1.50–4.60	3.51	0.65	1.80–5.00	0.05	0.829	0.00
Big Five Aspects Scale: Enthusiasm (Facet)	3.73	0.71	1.60–5.00	3.70	0.73	1.40–5.00	0.05	0.824	0.00
Big Five Aspects Scale: Assertiveness (Facet)	3.53	0.69	1.80–5.00	3.41	0.73	1.70–4.90	0.88	0.349	0.00
Big Five Aspects Scale: Intellect (Facet)	3.82	0.62	2.10–5.00	3.89	0.67	1.70–5.00	0.12	0.728	0.00
Big Five Aspects Scale: Openness (Facet)	3.77	0.73	1.50–5.00	3.82	0.66	1.50–5.00	0.09	0.769	0.00
BIS/BAS: Drive	11.43	2.52	5.00–16.00	10.08	2.42	5.00–16.00	11.80	<0.001	0.05
BIS/BAS: Funseeking	12.23	2.37	4.00–16.00	11.16	2.20	5.00–15.00	7.27	<0.01	0.03
BIS/BAS Reward Responsiveness	17.56	2.24	10.00–20.00	16.57	2.00	11.00–20.00	9.41	<0.005	0.04
BIS/BAS: BIS	21.66	3.88	11.00–28.00	19.62	3.54	8.00–26.00	18.35	<0.001	0.07
Interpersonal Reactivity Index: Perspective Taking	2.69	0.58	1.14–3.86	2.81	0.59	0.86–4.00	1.54	0.22	0.01
Interpersonal Reactivity Index: Fantasy	2.67	0.68	1.00–4.00	2.22	0.83	0.00–4.00	21.48	<0.001	0.08
Interpersonal Reactivity Index: Empathic Concern	2.87	0.53	1.43–3.86	3.07	0.52	1.57–4.00	6.85	<0.01	0.03
Interpersonal Reactivity Index: Personal Distress	1.55	0.61	0.00–2.86	1.10	0.57	0.00–2.57	27.27	<0.001	0.10
NIH Anger Affect	48.86	7.92	28.60–71.10	45.19	7.42	28.60–64.10	11.50	<0.001	0.04
NIH Anger Hostility	55.33	7.43	36.60–69.70	44.37	8.30	36.60–74.80	98.31	<0.001	0.28
NIH Anger Physical Aggression	56.03	9.18	43.40–79.20	47.79	7.05	43.40–68.80	52.76	<0.001	0.18
NIH Emotional Support	50.63	8.83	27.80–62.50	47.09	8.22	21.80–62.50	8.41	<0.005	0.03
NIH Fear Affect	55.88	6.96	32.90–74.70	50.30	7.58	32.90–84.90	29.67	<0.001	0.11
NIH Fear Somatic Arousal	55.04	9.73	40.10–81.40	46.68	5.95	40.10–62.80	51.51	<0.001	0.17
NIH Friendship	51.22	8.63	28.70–66.50	48.07	9.13	23.80–66.50	4.27	<0.05	0.02
NIH General Life Satisfaction	54.84	8.40	33.30–74.60	57.36	8.09	19.10–74.60	4.07	<0.05	0.02
NIH Instrumental Support	47.37	7.12	29.10–62.90	48.91	11.09	22.10–62.90	0.48	0.491	0.00
NIH Loneliness	54.50	8.49	37.60–73.90	50.39	7.63	37.60–74.60	10.97	<0.005	0.04
NIH Meaning and Purpose	49.25	8.83	27.60–71.60	51.43	9.12	26.20–71.60	2.25	0.135	0.01
NIH Perceived Hostility	51.08	7.48	33.50–68.80	48.56	7.78	33.50–69.50	9.65	<0.005	0.04
NIH Perceived Rejection	51.09	7.41	35.90–73.80	49.50	8.13	35.90–73.70	2.58	0.117	0.01
NIH Perceived Stress	52.43	9.45	26.40–76.20	43.53	8.99	26.30–82.00	48.37	<0.001	0.16
NIH Positive Affect	48.47	7.44	31.20–71.60	48.91	6.63	26.90–65.20	0.29	0.588	0.00
NIH Sadness	49.50	7.63	34.20–69.10	44.99	7.29	34.20–69.50	17.97	<0.001	0.07
NIH Self-Efficacy	49.08	8.25	32.30–68.40	51.88	9.52	0.00–68.40	4.58	<0.05	0.02

*Note*. NIH Emotion scores reflect T-scores. One-way ANCOVAs were conducted on each measure to test for age group differences with site, gender, and education as covariates. BIS/BAS = Behavioral Inhibition System/Behavioral Activation System.

### T1-MPRAGE

Cortical reconstruction and volumetric segmentation was performed with FreeSurfer version 6.0.1^41,42^. All participant surfaces had a Euler number of 2, indicating that no holes or defects were detected across the entire sample. Estimated total intracranial volume (eTIV), grey matter, white matter, hippocampus, BA45, V1, and MT volumes were extracted. These regions were selected for divergent susceptibilities to age-related volume reductions. Heteromodal cortices (prefrontal BA 45) and hippocampus characteristically show significant age-related volume losses, while volumes in unimodal cortices (V1, MT) are comparatively preserved into older age (^43–47^and see^8^, for a review). Regional volumes were adjusted for head size by using the residuals of a linear regression between each volume, as output by FreeSurfer, and eTIV^48–50^. Adjusted volumes for each hemisphere were summed to yield a single adjusted volume for each region. Estimated whole brain volume (eWBV) was also calculated as (grey matter + white matter)/eTIV. A series of ANCOVAs were then conducted to test for age group differences on volume with site, gender, education, and eWBV (regional volumes only) as covariates. Inclusion of eWBV provides additional estimation of specificity, particularly given global atrophy that occurs with aging (e.g.^51^,). Education was not recorded for 14 young adult participants.

**Fig. 1:**
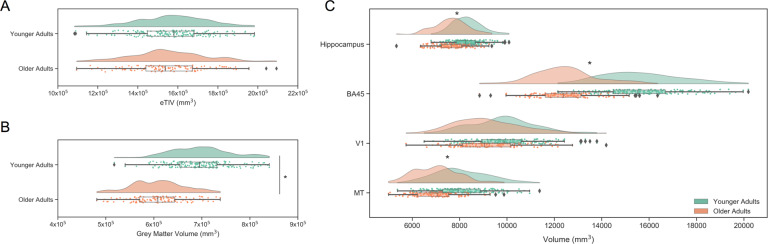
Structural MRI MPRAGE technical validation. (**A**) Estimated total intracranial volume (eTIV) in younger and older adults. (**B**) Grey matter volume in younger and older adults. (**C**) Hippocampal, lateral prefrontal (BA45), primary visual cortex (V1) and Motion Complex (MT) volumes in younger and older adults. Regional volumes were adjusted for eTIV. * indicates significant age group differences as determined by ANCOVAs controlling for site, gender, education, and eWBV (regional volumes only).

Volume distributions are plotted by age group in Fig. 1. Younger and older adults had comparable head sizes (Fig. 1A; *F*(1, 282) = 0.08, *p* = 0.784, η_p_^2^ = 0.00), but younger adults had higher grey matter volume (Fig. 1B; *F*(1,282) = 165.58, *p* < 0.001, η_p_^2^ = 0.37). Fig. 1C illustrates higher regional volumes in the hippocampus (*F*(1,281) = 12.60, *p* < 0.001, η_p_^2^ = 0.04), BA45 (*F*(1,281) = 116.67, *p* < 0.001, η_p_^2^ = 0.29), and MT (*F*(1,281) = 20.06, *p* < 0.001, η_p_^2^ = 0.07) in younger adults compared to older adults. V1 volumes were similar in younger and older adults (*F*(1,281) = 0.54, *p* = 0.463, η_p_^2^ = 0.00).

### T2-FLAIR

**Fig. 2:**
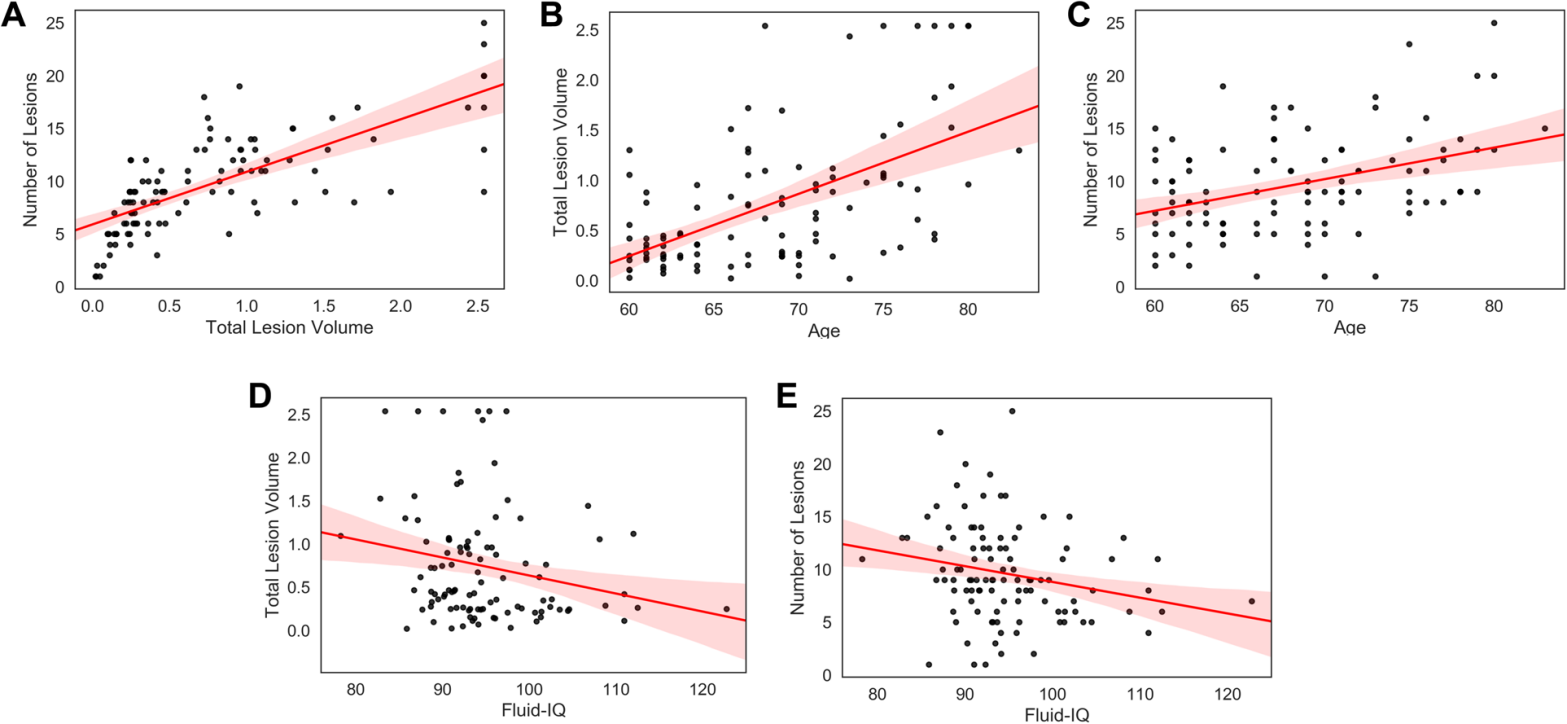
Technical validation of FLAIR images and white matter hyperintensities (**A**) Total lesion volume is associated with number of lesions. Increasing age is associated with (**B**) Total lesion volume, and, (**C**) Number of lesions. Fluid-IQ is negatively associated with (**D**) Total lesion volume, and, (**E**) Number of lesions. Total lesion volume is corrected for intracranial volume.

T2-FLAIR sequences were used to evaluate white matter hyperintensities (WMH) in 105 healthy older adults (57% female; *M*_*age*_ = 68.35; age range = 60–83 y). WMH were segmented by the lesion prediction algorithm (Schmidt, 2017, Chapter 6.1) as implemented in the Lesion Segmentation Toolbox version 2.0.15 (www.statistical-modelling.de/lst.html) for Statistical Parametric Mapping. Each participant’s raw total lesion volume in cubic millimetres (mm^3^) was then divided by their eTIV derived from the T1 image in mm^3^ to correct for head size. Final total lesion volume and number of lesions data were converted to within-sample z-scores for subsequent analysis. We characterized white matter lesion load, indexed by total lesion volume and number of lesions, and examined the validity of these indices to confirm that estimates of white matter lesion load in our sample demonstrated patterns previously established in the literature^43,52–55^. This was accomplished by examining white matter lesion load associations with age and cognition. Consistent with expectation, total lesion volume and number of lesions were positively correlated (*r*(103) = 0.730, *p* < 0.001, [0.60, 0.83]). White matter lesion load was then examined for its associations with age and cognitive performance. Both total lesion volume and number of lesions were positively correlated with age (*r*(103) = 0.562, *p* < 0.001, [0.42, 0.68]; *r*(103) = 0.398, *p* < 0.001, [0.23, 0.55], respectively). Both total lesion volume and number of lesions were negatively associated with the NIH fluid IQ composite score (*r*(101) = −0.220, *p = *0.013, [−0.36, −0.05]; *r*(101) = −0.227, *p = *0.011, [−0.37, −0.07], respectively). The associations between white matter lesion load and fluid IQ held when controlling for site, gender, age and education (total lesion volume: *pr*(97) = −0.179, *p = *0.038, [−0.34, 0.03]; number of lesions: *pr*(97) = −0.017, *p = *0.046, [−0.32, −0.02]. See Fig. 2.

### Resting-state ME-fMRI

To assess fMRI scan quality, quality metrics were calculated for each scan. Functional images were preprocessed with ME-ICA (version 3.2 beta^56,57^).

#### Framewise Displacement (FD)

A measure of the frame-to-frame movement, assessed in millimetres. FD was calculated on the second echo image for each resting-state scan using weighted scaling^38^. In younger adults, the average FD was 0.10 mm (*SD* = 0.05); in older adults, the average FD was 0.13 mm (*SD* = 0.05 mm).

#### Temporal Signal to Noise Ratio (tSNR)

**Fig. 3:**
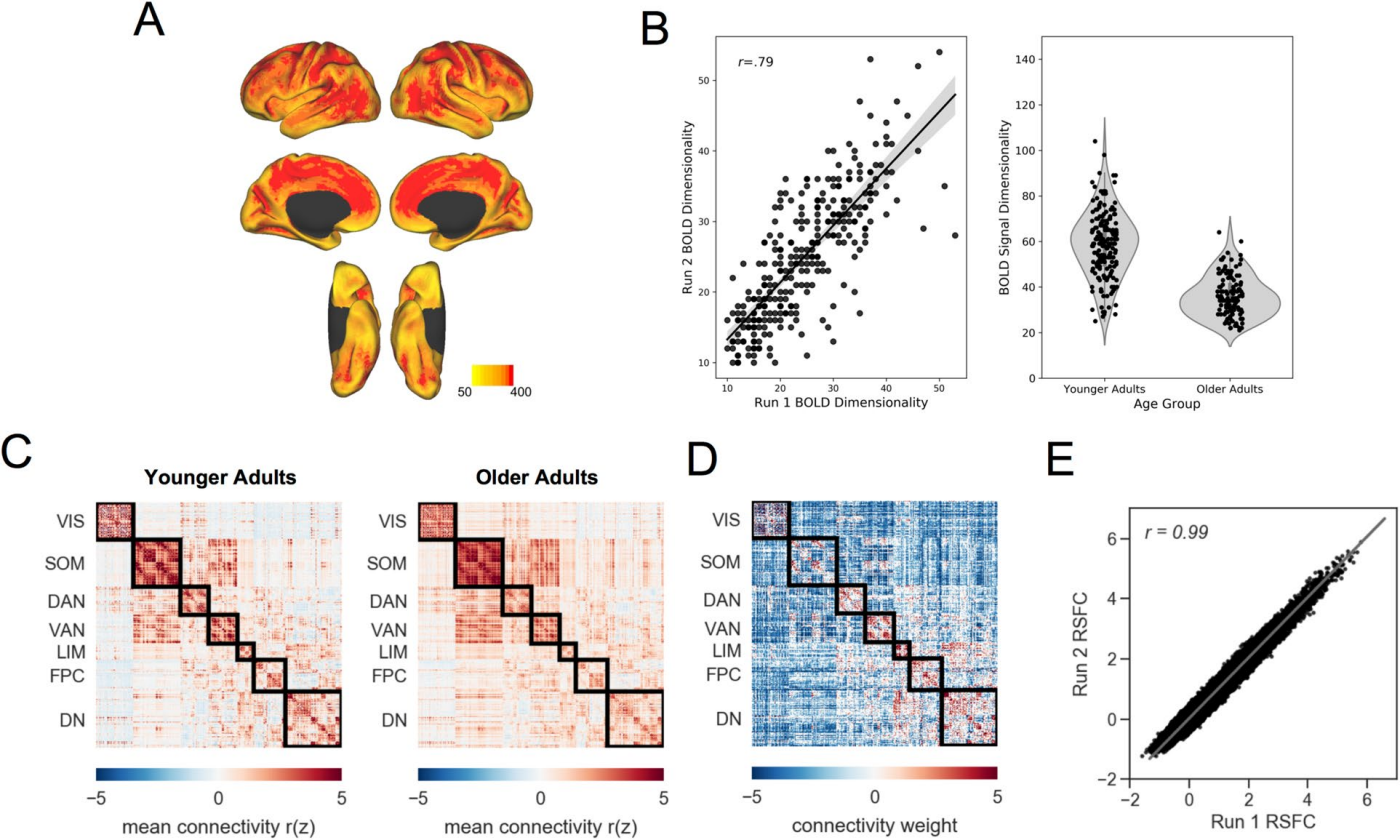
Functional MRI technical validation. (**A**) Temporal signal-to-noise map across the full sample. (**B**) BOLD signal dimensionality across runs (left) and in younger and older adults. (**C**) Resting-state functional connectivity for younger (left) and older (middle) adults. (**D**) Age-related differences in connectivity between younger and older adults. Red color indicates significantly greater connectivity in younger adults, and blue color indicates significantly greater connectivity in older adults. (**E**) Resting-state functional connectivity across runs (sample mean edge-weights). VIS = visual, SOM = somatomotor, DAN = dorsal attention, VAN = ventral attention, LIM = limbic, FPC = frontoparietal control, DN = default, RSFC = resting-state functional connectivity.

A measure of signal strength at the voxel level, calculated as the mean signal intensity of a voxel across the timeseries divided by its standard deviation. tSNR was calculated on each run of ME-ICA denoised data in native space (see^17^ for a comparison of single-echo and multi-echo). Following Kundu and colleagues^56^, tSNR was quantified within the conjunction of grey matter and functional masks. Skull-stripped anatomical images were resampled to functional resolution and segmented with FSL FAST to create the grey matter mask. AFNI 3dAutomask was applied to the functional data to create the functional mask. The median of all voxels within this mask is used to characterize each run of resting-state fMRI, where higher tSNR values reflect clearer signal. Median tSNR values for younger adults ranged from 140.10–361.37, and for older adults from 129.59–418.90. For visualization purposes, tSNR spatial maps were separately derived in standard MNI space across the whole brain. Maps were averaged across all participants, thresholded at 50, and plotted in Fig. 3A. The results clearly demonstrate high tSNR throughout the cortical mantle in both cohorts.

#### BOLD dimensionality

A unique advantage of ME-fMRI and the ME-ICA processing framework is that BOLD and non-BOLD signals can be separated into independent components. A novel metric of “BOLD dimensionality”^58^, or the number of BOLD components identified in the ME-fMRI timeseries, may then be examined. We assessed test-retest reliability of BOLD dimensionality across two runs of data and compared BOLD dimensionality between younger and older adults. BOLD dimensionality was stable across resting-state fMRI runs (r(299) = 0.79, p < 0.001, [0.75, 0.83]; Fig. 3B left panel). Younger adults showed greater BOLD dimensionality than older adults (*t*(299) = 15.38, *p* < 0.001, [20.06, 25.95], Cohen’s *d* = 1.81; Fig. 3B right panel). This remained true when controlling for site, gender, education, and eWBV (*F*(1,281) = 97.07, *p* < 0.001; η_p_^2^ = 0.26).

#### Connectomics

Whole-brain interregional functional connectivity was computed and compared between younger and older adults. Group mean connectivity matrices are in Fig. 3C. Age-related differences in the 79800 interregional connections (i.e., the lower triangle of the 400 × 400 functional connectivity matrix^59^; individualized with Group Prior Individualized Parcellations^60^) were quantitatively assessed with Partial Least Squares^61,62^. A significant latent variable (permuted *p* = 0.001) revealed a pattern of age differences in RSFC, with increases and decreases observed across the connectome (Fig. 3D). See Setton and Mwilambwe-Tshilobo *et al*.^7^ for in depth assessment.

## Data Availability

ME-ICA uses AFNI and python, both of which are open-source software. ME-ICA processing code is available at https://github.com/ME-ICA/me-ica. As the code base is unmaintained, readers are also directed to https://tedana.readthedocs.io/ for additional multi-echo de-noising options.
