# Author Correction: Neurocognitive aging data release with behavioral, structural and multi-echo functional MRI measures

**DOI:** 10.1038/s41597-024-03265-5

**Published:** 2024-04-24

**Authors:** R. Nathan Spreng, Roni Setton, Udi Alter, Benjamin N. Cassidy, Bri Darboh, Elizabeth DuPre, Karin Kantarovich, Amber W. Lockrow, Laetitia Mwilambwe-Tshilobo, Wen-Ming Luh, Prantik Kundu, Gary R. Turner

**Affiliations:** 1 Montreal Neurological Institute, Department of Neurology and Neurosurgery, McGill University, Montreal, QC Canada; 2 McConnell Brain Imaging Centre, McGill University, Montreal, QC Canada; 3 Departments of Psychiatry and Psychology, McGill University, Montreal, QC Canada; 4 Douglas Mental Health University Institute, Verdun, QC Canada; 5 Department of Psychology, York University, Toronto, ON Canada; 6 Department of Psychology, Ryerson University, Toronto, ON Canada; 7 Department of Psychiatry, University of Toronto, Toronto, ON Canada; 8 National Institute on Aging, National Institutes of Health, Baltimore, MD USA; 9 Icahn School of Medicine at Mount Sinai, New York, NY USA

**Keywords:** Cognitive neuroscience, Cognitive ageing, Magnetic resonance imaging, Cognitive neuroscience, Cognitive ageing, Magnetic resonance imaging

Correction to: *Scientific Data* 10.1038/s41597-022-01231-7, published online 29 March 2022

The authors were alerted to the fact that the original data for Tails B minus A was coded inconsistently across data collection sites. One site used seconds, the other, milliseconds, which was later corrected in the data file at OSF (http://osf.io/yhzxe/). These changes have been documented at OSF via a changelog (https://osf.io/3q6fs). Because of this, the descriptive statistics in tables 1 and 2 were incorrect in the original version as they were based on the original data. These have been recalculated using the rescaled data and corrected in the pdf and HTML versions of the paper. The authors thank Michael Truong, Tarnpreet Virk, and Shir Bach-Kay for their diligence and communication regarding this error.

